# Enhanced chickpea (*Cicer arietinum* L.) growth and yield attributes via agronomic modulation using nano-emulsion biofertilizer for sustainable crop productivity improvement

**DOI:** 10.3389/fmicb.2026.1795467

**Published:** 2026-05-18

**Authors:** M. Sri Abisankar, R. Augustine, S. P. Jeshwin Giftson, R. S. Graceson, P. Ramesh Kumar, K. Indira Petchiammal, S. Praveena Katharine, T. Dhivyalakshmi, R. Santhosh Anto Kumar

**Affiliations:** 1 Division of Agronomy, School of Agricultural Sciences, Karunya Institute of Technology and Sciences, Coimbatore, Tamil Nadu, India; 2 Division of Crop Physiology and Biochemistry, School of Agricultural Sciences, Karunya Institute of Technology and Sciences, Coimbatore, Tamil Nadu, India; 3 Division of Genetics and Plant Breeding, School of Agricultural Sciences, Karunya Institute of Technology and Sciences, Coimbatore, Tamil Nadu, India; 4 Division of Soil Science, School of Agricultural Sciences, Karunya Institute of Technology and Sciences, Coimbatore, Tamil Nadu, India

**Keywords:** chickpea, nano fertilizer, nano-emulsion biofertilizer, NPK, soil and foliar application, nutrient management

## Abstract

Chickpea (*Cicer arietinum* L.) productivity is often constrained by inefficient nutrient management and declining soil fertility. This study evaluated the potential of integrating reduced mineral fertilization with nano-emulsion biofertilizers (NEB) to enhance growth, yield, and grain quality under field conditions. A two-factor field experiment was conducted during 2023–2024 at the Karunya Institute of Technology and Sciences, India. Treatments comprised four NPK levels (0, 50, 75, and 100% of 25:50:20 kg ha^−1^) combined with foliar applications of nano fertilizers ND (Nano-DAP), NU (Nano-Urea) and NEB (Nano Emulsion Biofertilizer; Rhizobium + Phosphorus Solubilizing Bacteria (PSB) consortium) applied at 30 and 45 days after sowing. Results revealed that 75% NPK integrated with NEB significantly improved growth traits, including plant height, number of branches, leaf area index, chlorophyll content, and dry matter production. Yield attributes such as pods per plant, grains per plant, and 100-seed weight were markedly enhanced, resulting in the highest grain yield (1143.97 kg ha^−1^) and stover yield (2024.09 kg ha^−1^), the combined application of 75% NPK + NEB increased grain yield by an average of 45.3% compared with the other treatments. Grain quality also improved significantly, with higher protein content (28.69%) and increased zinc and iron concentrations. Correlation and structural equation modeling indicated strong positive relationships between growth parameters and yield (R^2^ = 0.80), with dry matter production and leaf area index acting as key determinants. The study demonstrates that substituting 75% of conventional fertilizers with NEB is an efficient and sustainable strategy to improve nutrient use efficiency, crop productivity, and grain nutritional quality, supporting environmentally resilient chickpea production system.

## 1 Introduction

Globally, pulses play a vital role in human nutrition, particularly in regions affected by protein and micronutrient deficiencies, where they serve as a primary source of dietary protein and essential nutrients. In addition to their protein richness, pulses contain slowly digestible starch, a desirable carbohydrate fraction that is gradually hydrolyzed in the small intestine, thereby reducing postprandial blood glucose and insulin responses (Jenkins et al., 1981). Although starch constitutes approximately 35–60% of pulse composition, these crops are generally valued more for their protein content than as carbohydrate sources (Deshpande et al., 1982). However, the extraction of high-purity starch from pulses remains technically challenging due to the presence of highly hydrated fiber fractions and insoluble proteins that interfere with separation processes (Schoch and Maywald, 1968).

**Figure 1:**
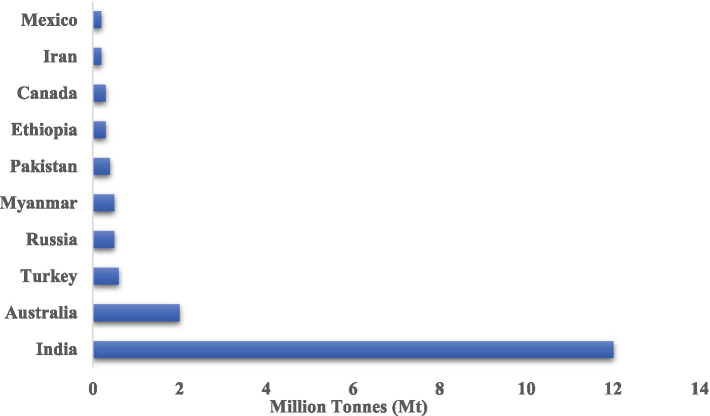
Region-wise distribution and top 10 chickpea producing countries in year 2024 (million tonnes, Mt).

Chickpea (*Cicer arietinum L.*) is a globally significant legume valued for its high protein content, ability to improve soil fertility through biological nitrogen fixation, and adaptability to semi-arid agro-ecological conditions. However, achieving optimal productivity and sustainability in chickpea cultivation remains challenging due to various abiotic stresses and agronomic constraints, including nutrient-deficient soils, salinity, and water scarcity. According to FAO (Food and Agriculture Organization of the United Nations) (2024), India is the leading producer of chickpea globally, primarily due to its large cultivation area, while Australia ranks second with relatively higher productivity owing to efficient crop management practices (Figure 1).

Conventional fertilizer application methods are often characterized by low nutrient use efficiency and can lead to environmental degradation through nutrient losses such as leaching and volatilization. These limitations have driven the search for sustainable nutrient management strategies that can enhance crop productivity while minimizing ecological impacts. In this context, nano-emulsion biofertilizers, which combine nanotechnology with beneficial microorganisms such as Rhizobium and phosphorus solubilizing bacteria (PSB), have emerged as an innovative approach for efficient nutrient delivery (Cathrine Christina et al., 2025; Sri Abisankar et al., 2024). These formulations improve root nutrient absorption and reduce nutrient losses, thereby enhancing overall nutrient use efficiency (Oyediran et al., 2025; Garg et al., 2023; Phiri et al., 2023; Arriagada et al., 2022).

Previous studies have demonstrated that nano-emulsion-based fertilizers can significantly improve vegetative growth and yield components in chickpea, including pod number and seed weight, while also enhancing tolerance to abiotic stresses. By stimulating microbial activity in the rhizosphere, these formulations facilitate faster nutrient cycling, leading to improved soil fertility, plant health, and productivity, thereby aligning with the principles of sustainable agriculture. Nevertheless, the large-scale adoption of nano-emulsion fertilizers is constrained by factors such as high production costs, regulatory challenges, and uncertainties regarding long-term environmental effects, which require further investigation and policy support (Manish et al., 2024).

Nanotechnology has broad applications in agriculture, including the development of nano-based formulations for crop protection, pest and disease detection using nano sensors, and improved efficiency (Ghidan and Al Antary, 2019). In addition, precision agriculture approaches have demonstrated potential in reducing nutrient losses, particularly nitrogen losses through leaching, volatilization, and microbial processes in soil (Wang et al., 2022). The application of nanotechnology extends across the entire agricultural value chain, from field production to storage, transportation, and marketing (Tan et al., 2019). However, despite its potential benefits, concerns regarding environmental safety and associated risks necessitate comprehensive risk assessment frameworks before widespread adoption.

A growing body of research indicates that nano-fertilizers can enhance nutrient uptake efficiency, reduce soil toxicity, and improve crop productivity compared to conventional fertilizers (Raliya et al., 2017; Deng et al., 2020; Wang et al., 2021). These inputs can minimize fertilizer wastage, lower input costs, and support sustainable agricultural practices. Furthermore, combining nano-fertilizers with bio-compatible nanomaterials and beneficial microorganisms has led to the development of advanced biofertilizer systems capable of delivering nutrients in a controlled and sustained manner throughout the crop growth cycle. This ensures improved nutrient availability, enhanced uptake efficiency, and ultimately higher crop yields (Thirugnanasambandan, 2018).

Nano-emulsion biofertilizers are developed by integrating beneficial microbial strains such as rhizobium and phosphorus-solubilizing bacteria with nanoscale carrier systems, which enhance microbial survival and enable targeted nutrient delivery (Oyediran et al., 2025; Cathrine Christina et al., 2025). The use of nano-formulations, including nano-DAP and nano-NPK, for seed treatment and foliar application further improves nutrient uptake, root colonization, and balanced plant nutrition (Ram et al., 2025; Meena D. et al., 2024). Integrating reduced doses of conventional fertilizers with nano-emulsion biofertilizers represents an efficient strategy to maximize resource utilization without compromising crop productivity. Strategic timing of applications, including seed priming and foliar sprays at critical growth stages, enhances physiological responses such as plant height, branching, pod development, and dry matter accumulation (Sarika et al., 2025).

Overall, the integration of inorganic fertilizers with nano-emulsion biofertilizers provides a sustainable, economically viable, and environmentally friendly approach for improving chickpea growth, yield, and soil health while reducing the negative impacts associated with excessive chemical fertilizer use.

In view of the above situations, this study was carried out to demonstrate the Enhanced Chickpea (*Cicer arietinum* L.). Growth and Yield Attributes via Agronomic Modulation Using Nano-Emulsion Biofertilizer for Sustainable Crop Productivity Improvement, with the following objectives:

1. To study the influence of the nutrient sources on the crop growth and yield of chickpea.
2. To optimize the use of effective soil and foliar nutrition for enhancing the growth and yield of chickpea.

## 2 Materials and methods

### 2.1 Experimental conditions and treatments

The field experiment was conducted during 2023 and 2024 at South Farm, School of Agricultural Sciences (SAS), Karunya Institute of Technology and Sciences (KITS), India (10.94° N, 76.75° E; 467 m above mean sea level). The experimental soil was silty clay loam with 5.40% organic matter (0–20 cm), available N (180 kg ha^−1^), P (13.7 kg ha^−1^), K (298 kg ha^−1^), pH 8.3, and EC 0.37 dS m^−1^, determined following standard soil analysis procedures (Jackson, 1973; Olsen and Sommers, 1982).

**Figure 2:**
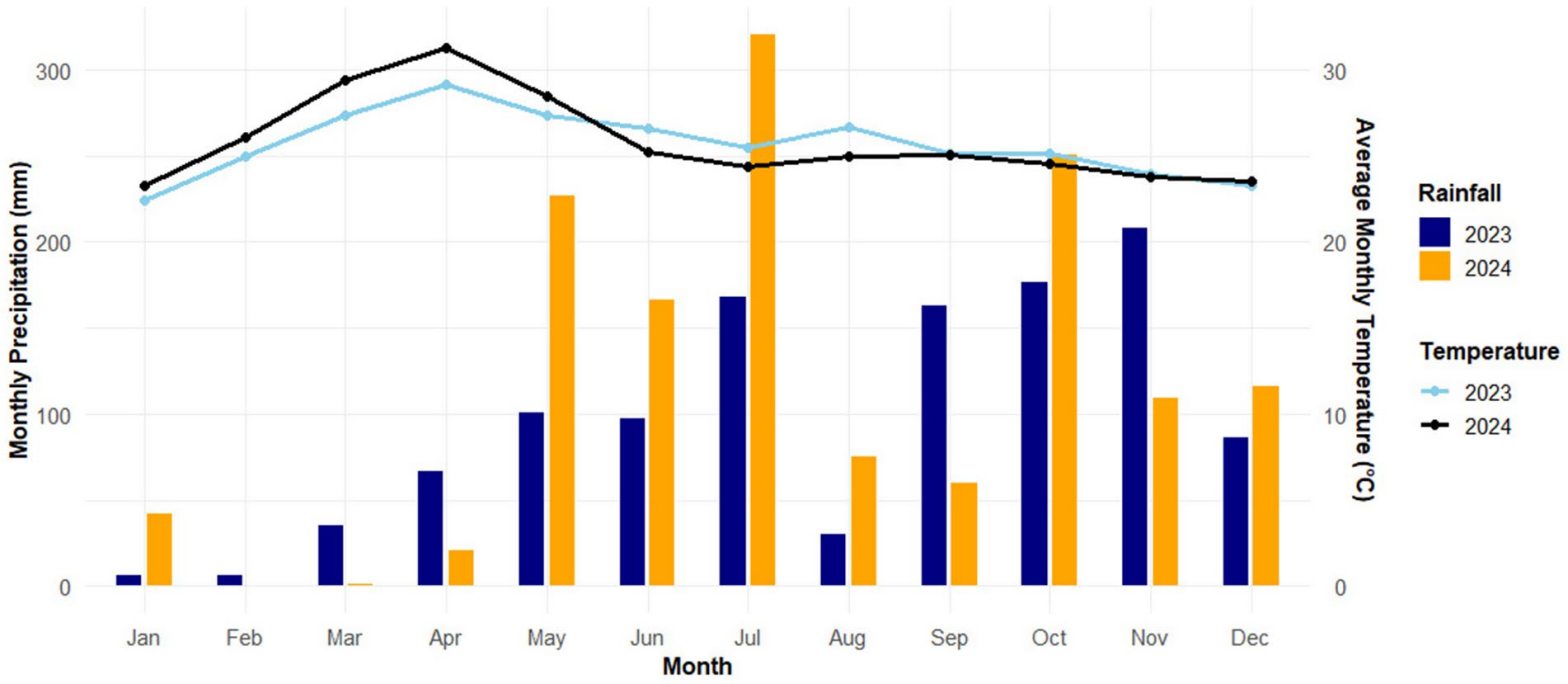
Temperature, **r**ainfall, and precipitation during the growing seasons of 2023 and 2024, according to the meteorological post of the KITS Agricultural Experiment Station.

The weather conditions during the crop seasons varied considerably. Annual precipitation during the crop period was 270.5 mm (2023) and 297.0 mm (2024), exceeding the long-term average by 12.71 and 23.75%, respectively (Figure 2).

The treatments consisted of five levels of mineral fertilization: N₀P₀K₀, N₂₅P₅₀K₂₀, N₁₈.₇₅P₃₇.₅K₁₅, N₁₂.₅P₂₅K₁₀, and N₁₀P₂₀K₈, combined with nano fertilizers (Nano DAP (ND) and Nano Urea (NU)) and Nano emulsion biofertilizer (NEB) treatments (Table 1). The experiment followed a randomized block design with two factorial arrangement (Gomez and Gomez, 1984).

**Table 1:** Design of the experimental treatments.

Treatment details	
First factor (S)	Soil fertilization
S_1_: No Fertilizers (NF)	N_0_ P_0_ K_0_
S_2_: 100% NPK	N_25_ P_50_ K_20_
S_3_: 75% NPK	N_18.75_ P_37.5_ K_15_
S_4_: 50% NPK	N_12.5_ P_25_ K_10_
Second factor (F)	Foliar fertilization
F_1_: Nano-DAP (ND)	2 foliar sprays @ 0.2% @ 30 and 45 DAS
F_2_: Nano-Urea (NU)	2 foliar sprays @ 0.2% @ 30 and 45 DA
F_3_: Nano-emulsion biofertilizer (NEB)	2 foliar sprays @ 10 mL l^−1^ @ 30 and 45 DAS
Treatment combination	
S_1_F_1_: NF + ND	N_0_ P_0_ K_0_ + Nano DAP (2 foliar sprays @ 0.2% @ 30 and 45 DAS)
S_1_F_2_: NF + NU	N_0_ P_0_ K_0_ + Nano Urea (2 foliar sprays @ 0.2% @ 30 and 45 DAS)
S_1_F_3_: NF + NEB	N_0_ P_0_ K_0_ + Nano Emulsion Biofertilizer (2 foliar sprays @ 10 mL l^−1^ @ 30 and 45 DAS)
S_2_F_1_: 100% NPK + ND	N_25_ P_50_ K_20_ + Nano DAP (2 foliar sprays @ 0.2% @ 30 and 45 DAS)
S_2_F_2_: 100% NPK + NU	N_25_ P_50_ K_20_ + Nano Urea (2 foliar sprays @ 0.2% @ 30 and 45 DAS)
S_2_F_3_: 100% NPK + NEB	N_25_ P_50_ K_20_ + Nano Emulsion Biofertilizer (2 foliar sprays @ 10 mL l^−1^ @ 30 and 45 DAS)
S_3_F_1_: 75% NPK + ND	N_18.75_ P_37.5_ K_15_ + Nano DAP (2 foliar sprays @ 0.2% @ 30 and 45 DAS)
S_3_F_2_: 75% NPK + NU	N_18.75_ P_37.5_ K_15_ + Nano Urea (2 foliar sprays @ 0.2% @ 30 and 45 DAS)
S_3_F_3_: 75% NPK + NEB	N_18.75_ P_37.5_ K_15_ + Nano Emulsion Biofertilizer (2 foliar sprays @ 10 mL l^−1^ @ 30 and 45 DAS)
S_4_F_1_: 50% NPK + ND	N_12.5_ P_25_ K_10_ + Nano DAP (2 foliar sprays @ 0.2% @ 30 and 45 DAS)
S_4_F_2_: 50% NPK + NU	N_12.5_ P_25_ K_10_ + Nano Urea (2 foliar sprays @ 0.2% @ 30 and 45 DAS)
S_4_F_3_: 50% NPK + NEB	N_12.5_ P_25_ K_10_ + Nano Emulsion Biofertilizer (2 foliar sprays @ 10 mL l^−1^ @ 30 and 45 DAS)

The seed of chickpea, variety NBeG-49 were used for this study. The sowing of chickpea was done at a row spacing of 30 cm and plant to plant with 10 cm. Seeds were dibbled at of two seed hill^−1^.

Basal application of phosphorus (50 kg P₂O₅ ha^−1^ as Single Super Phosphate) and potassium (20 kg K₂O ha^−1^ as Muriate of Potash) was done prior to sowing, while nitrogen (25 kg ha^−1^ as Urea) was applied as pre-sowing fertilizer following recommended agronomic practices (Indian Council of Agricultural Research (ICAR), 2018). Chickpea was sown in the 1st fortnight of October at a depth of 6–8 cm with a seed rate equivalent to 300 viable seeds m^−2^.

Foliar application of ND, NU, and NEB (consortium of PSB and Rhizobium) at 10 mL L^−1^ was carried out at flowering and pod formation stages (30 and 45 DAS), following standard foliar nutrition protocols (Fageria, 2001). All other agronomic practices were maintained as per recommended package of practices for chickpea cultivation (Indian Council of Agricultural Research (ICAR), 2018).

### 2.2 Soil sampling and testing

Soil samples (0–20 cm depth) were collected before sowing using an auger in a zig-zag pattern to form a composite sample. Samples were air-dried, ground, and passed through a 2-mm sieve prior to analysis. Soil physicochemical properties including p^H^, organic carbon (OC%), available nutrients (NPK, kg ha^−1^), and C: N ratio were determined using standard analytical procedures (Jackson, 1973; Page et al., 1982). Available nutrients were extracted using the Mehlich-3 method (Mehlich, 1978).

### 2.3 Growth components

Plant height and number of branches per plant were recorded from 10 randomly selected plants per plot at physiological maturity. Dry matter production (DMP) was determined by oven-drying plant samples at 75 ± 5 °C to constant weight [AOAC (Association of Official Analytical Chemists), 2005].

The formula for the Dry Matter Production (DMP):


$$\;\;\mathrm{DMP}=\frac{\begin{array}{l} \mathrm{Dry}\;\text{Weight of the Plant}\\ \times \text{Plant popoulation}\;{\mathrm{ha}}^{-1} \end{array}}{\begin{array}{c} \text{Number of samples taken for biomass}\;\\ \text{estimation}\;\mathrm{per}\;\text{treatments}\times 1000 \end{array}}$$

Leaf Area Index (LAI) was calculated using the standard formula:


$$\mathrm{LAI}=\frac{\text{Leaf Area}}{\text{Ground Area}\;(m^{2})\;}$$

Where Leaf Area = L × B × N × K.

L, Length of the leaf,

B, Breadth of the leaf,

N, Number of leaves,

K, Constant factor (0.75).

It is an important measure in ecology and agriculture used to evaluate plant canopy structure, light interception, and photosynthetic activity. This concept was introduced by Balakrishnan et al., 1987. Chlorophyll content was measured at 55 DAS using a SPAD-502 chlorophyll meter (Konica Minolta, Japan), following the non-destructive method described by Minolta Camera Co., Ltd. (1989).

### 2.4 Yield and yield components

Yield attributes such as number of pods per plant and grains per plant were recorded from 10 randomly selected plants per plot. The crop was harvested at physiological maturity, and 100-seed weight was recorded at 10% moisture content following standard procedures [ISTA (International Seed Testing Association), 2023]. Grain yield, stover yield, and harvest index (HI) were calculated from the net plot area. Harvest index was computed as the ratio of grain yield to total above-ground biomass (Unkovich et al., 2010).

The formula for the Harvest Index (%):


$$\text{Harvest Index}\;(\%)=\frac{\text{Economical Yield}\;(\mathrm{kg}\;{\mathrm{ha}}^{-1})}{\text{Biological Yield}\;(\mathrm{kg}\;{\mathrm{ha}}^{-1})}\times 100$$

### 2.5 Grain nutrient analysis

Grain protein content was estimated using the Kjeldahl nitrogen method and converted using a formula, Protein (%) = N content (%) × 6.25 [AOAC (Association of Official Analytical Chemists), 2005; Parkinson and Allen, 1975]. Zinc and iron contents were determined after wet digestion (HNO₃ + HClO₄) using an Atomic Absorption Spectrophotometer (AAS) following standard protocols (Lindsay and Norvell, 1978).

### 2.6 Statistical analysis

Data were analyzed using analysis of variance (ANOVA) for a factorial randomized block design as described by Gomez and Gomez (1984). Pooled analysis over 2 years was performed due to homogeneity of variance. Treatment means were compared using F-test at *p* ≤ 0.05. Statistical computations were carried out using IBM SPSS Statistics (version 25).

## 3 Results

### 3.1 Plant height

**Figure 3:**
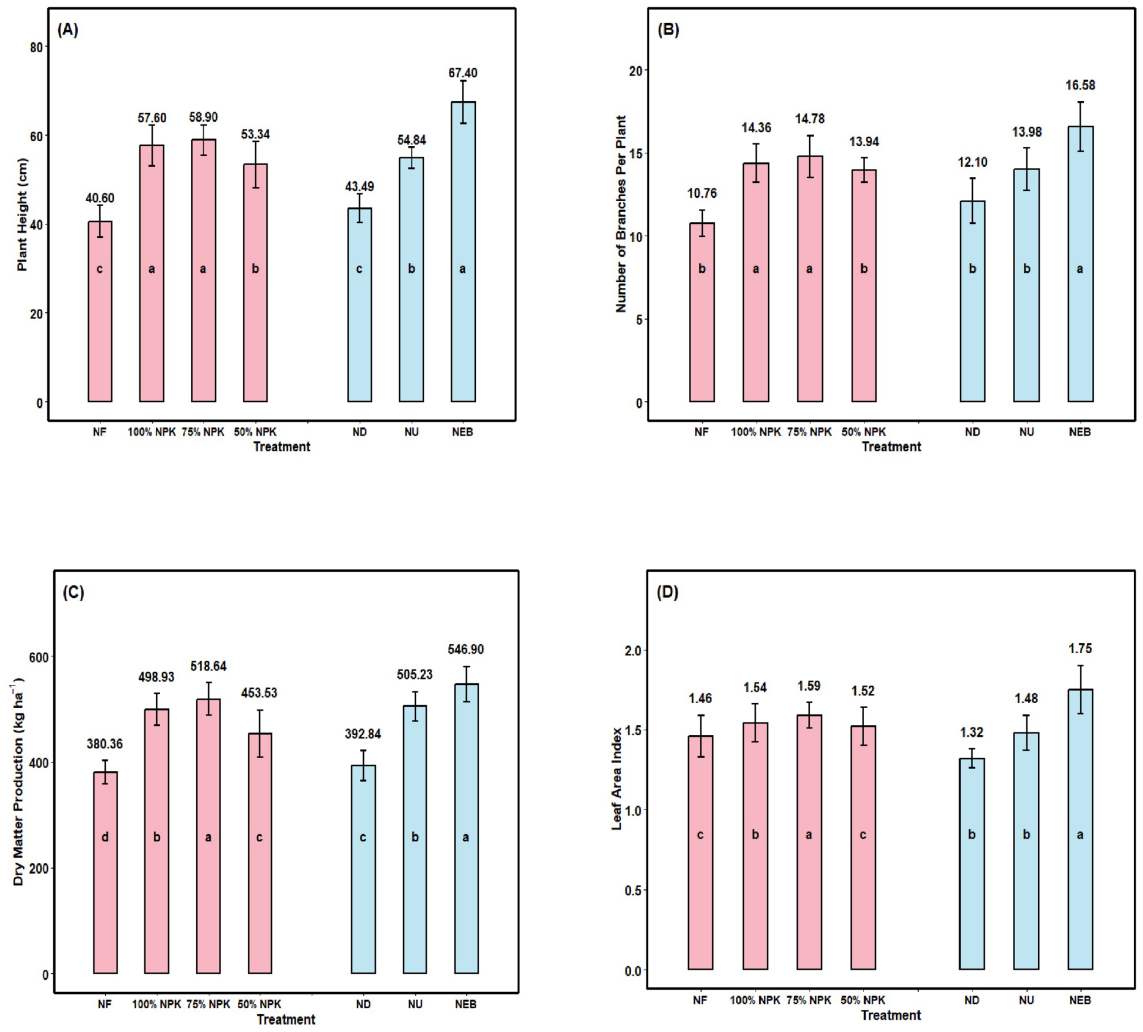
**(A)** Plant height (PH), **(B)** number of branches per plant (NBP), **(C)** dry matter production (DMP), and **(D)** leaf area index (LAI) of chickpea as influenced by mineral fertilization, foliar application, and their combination. NF—N_0_P_0_K_0_; 100% NPK—N_25_P_50_K_20_; 75% NPK—N_18.75_P_37.5_K_15_; 50% NPK—N_12.5_P_25_K_10_; ND—Nano DAP; NU—Nano Urea; NEB—Nano Emulsion Biofertilizer. Bars with different lowercase letters indicate significant differences among treatments (F-test, *p* ≤ 0.05). Error bars represent the standard error of the mean.

The findings demonstrate a substantial impact of mineral fertilization (75% NPK) on chickpea plant height (Figure 3A). It increased by an average of 45.1% following the 75% NPK treatment compared with the control. Depending on the microbial preparations and nano fertilizers, the height of the chickpea plant increased from 43.49 cm to 67.40 cm in the version that combined foliar plant nutrition with a nano-emulsion biofertilizer. Under the influence of the applied nano-emulsion biofertilizers, there was a statistically significant increase in chickpea plant height (Table 2).

**Table 2:** Results (*p*-values) of a two-way ANOVA of the effect of soil application, foliar plant application, and their interaction on the morphological features of plants, physiological traits, yield components, and yield.

Treatment	PH	NBP	DMP	LAI	CC	NP	NGP	TSW	GY	SY	HI	CP	Zn	Fe
S	0.66	0.03	6.64	0.002	1.54	0.38	1.4	1.51	13.01	33.66	0.33	0.93	0.43	0.57
F	0.85	0.04	8.57	0.002	1.99	0.49	1.89	1.95	16.80	43.45	0.42	1.20	0.56	0.73
S x F	1.48	0.06	14.85	0.004	3.44	0.84	3.27	3.37	29.10	75.26	0.73	2.09	0.97	1.27

Plant height (PH), number of branches per plant (NBP), dry matter production (DMP), leaf area index (LAI), chlorophyll content (CC), number of pods per plant (NP), number of grains per plant (NG), 100—seed weight (TSW), grain yield (GY), stover yield (SY), harvest index (HI), crude protein (CP), zinc content (Zn), iron content (Fe).

### 3.2 Number of branches per plant

The study’s findings show that 75% NPK fertilization significantly increased leaf production intensity per unit area in chickpea (Figure 3B). The number of branches per plant (NBP) increased by 37.4% in the 75% NPK-applied sites when compared to the version that did not receive mineral fertilization (NF). The value of NBP is significantly affected by the use of micronutrient preparations and nano-fertilizers. Compared with nano fertilizers, the value of nano-emulsion biofertilizer increased by almost 37%.

### 3.3 Dry matter production

The study found that the mineral fertilizers significantly reduced the amount of dry matter that chickpea plants accumulated (Figure 3C). In contrast to areas where mineral fertilizer was not applied, the DMP increased by an average of 28.92% in the 75% NPK applied versions. Compared with the ND and NU, the NEB significantly increased plant dry weight (98.41 and 36.84%, respectively), regardless of NPK fertilization. When it came to the chickpea plants’ ability to accumulate dry biomass, the NEB combination (Rhizobium + PSB) foliar spray proved to be the most effective. The maximum DMP amount per hectare was 632.03 kg ha^−1^ for this variety, and this amount was considerably greater than for any of the other two foliar treatments.

### 3.4 Leaf area index

Our results show the significant influence of the recommended NPK fertilizer rate on the amount of organic matter produced per chickpea leaf area (Figure 3D). In comparison to the non-mineral fertilized version (NF), the leaf area index (LAI) was significantly greater (8.90% greater) at areas that received 75% NPK. The foliar application of NEB had a considerable impact on LAI. There was a considerable increase in LAI in comparison to control, caused by the utilization of ND and NU, microbiological preparations (NEB), micro-fertilizers, and their combination.

### 3.5 Chlorophyll content

**Figure 4:**
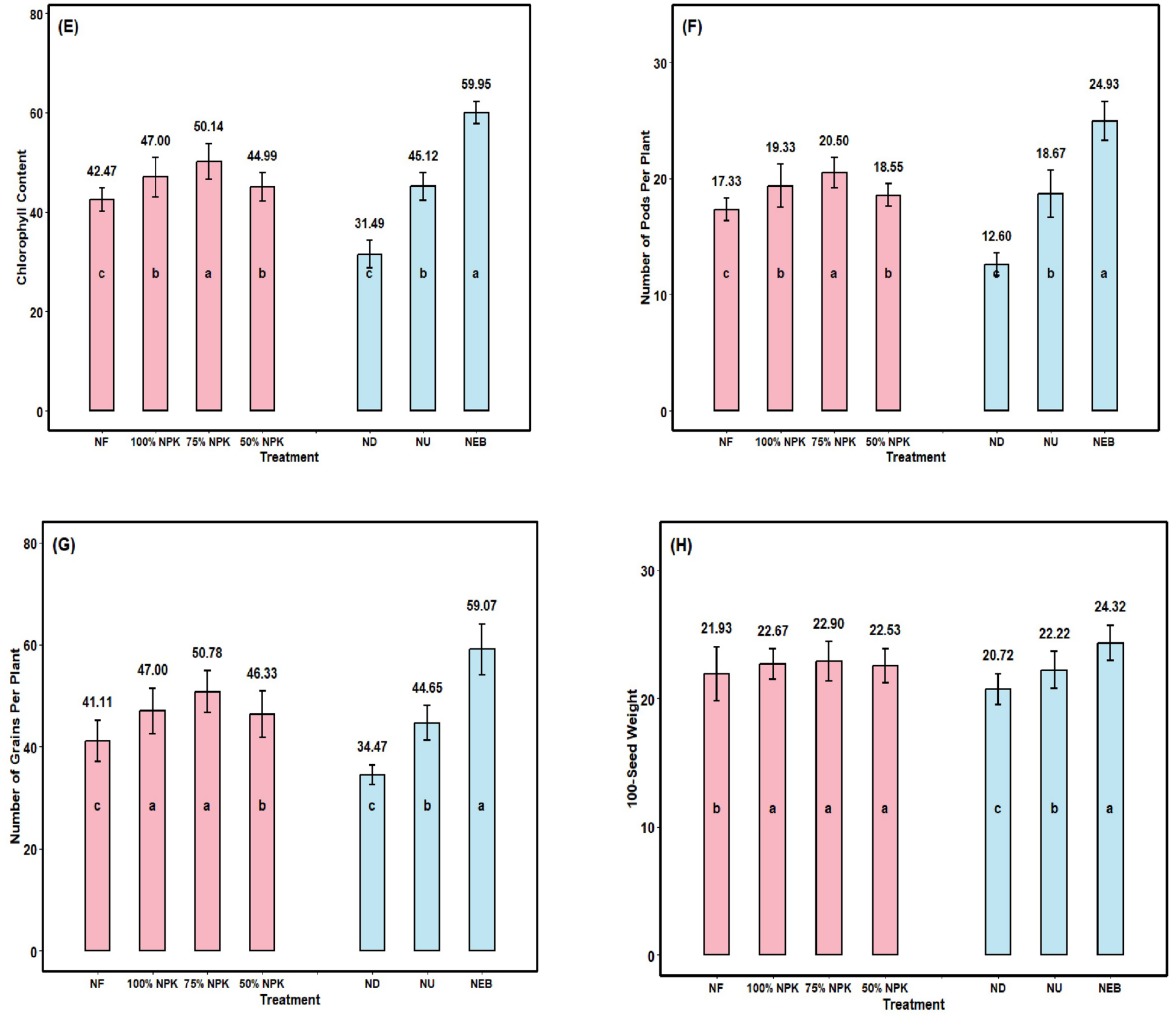
**(E)** Chlorophyll content (CC), **(F)** number of pod per plant (NP), **(G)** number of grains per plant (NG), and **(H)** 100-seed weight (TSW) of chickpea depending on mineral fertilization, foliar application, and their combination; NF—N_0_ P_0_ K_0_; 100%NPK—N_25_ P_50_ K_20_; 75%NPK—N_18.75_ P_37.5_ K_15_; 50%NPK—N_12.5_ P_25_ K_10_; ND—Nano DAP; NU—Nano Urea; NEB—Nano Emulsion Biofertilizer. Bars with different lowercase letters indicate significant differences among treatments (F-test, *p* ≤ 0.05). Error bars represent the standard error of the mean.

As one of the key factors influencing yield, an increase in the plants’ CC led to higher LAI and DMP (Figure 4E). Compared to the NF object, the CC rose by 18.1% in the NPK application variations. While the nano fertilizer and emulsion biofertilizer application variants tended to increase the CC, the NEB and NU versions showed a considerable increase in the CC relative to the control, by 90.40 and 43.3%, respectively.

### 3.6 Number of pods per plant

Applications of mineral fertilizers and microbial treatments, applied singly or in tandem, significantly boosted key chickpea yield components. When compared to the NF version, the application of NPK significantly increased NP (by 12.25% on average; Figure 4F). The NP was altered to 12.60–24.93 pods per plant by the applied nano fertilizer and nano emulsion biofertilizer preparations, but only foliar feeding with NEB showed a significant increase of 97.86% compared to ND and 48.17% compared to NU.

### 3.7 Number of grains per plant

As the number of pods per plant (NP) serves as a primary yield determinant, elevated NP levels correspondingly boosted the number of grains per pod (NGP), as depicted in Figure 4G. The NGP rose by 23.52% in comparison to the NF object in the 75% NPK application versions. The nano-emulsion biofertilizer application variants showed a propensity to raise the NGP, while the NEB variants showed a considerable rise in the NGP (by 29.39 and 71.37%, respectively) when compared to the ND and NU controls.

### 3.8 100-seed weight (TSW)

The 100-seed weight increased following NPK application, thereby improving plant nutritional quality (Figure 4H). The average increase in this feature compared to the NF object was 3.51% in the NPK application variations, where it was most noticeable. Compared with the ND and NU, the foliar application of the NEB preparations significantly increased 100-seed weight by 7.34 and 17.49%, respectively.

### 3.9 Grain yield

**Figure 5:**
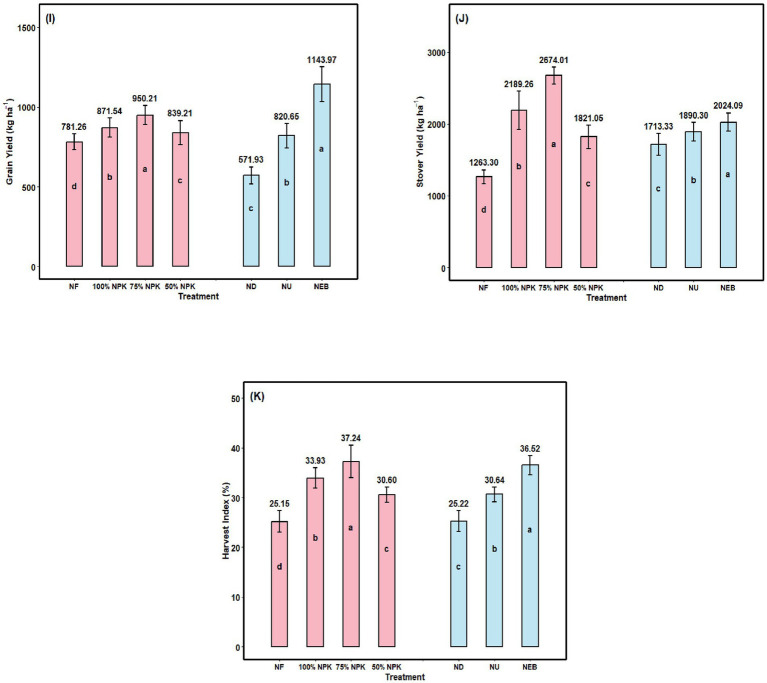
**(I)** Grain yield (GY), **(J)** Stover yield (SY), and **(K)** Harvest index (HI) of chickpea depending on mineral fertilization, foliar application, and their combination; NF—N_0_ P_0_ K_0_; 100% NPK—N_25_ P_50_ K_20_; 75%NPK—N_18.75_ P_37.5_ K_15_; 50%NPK—N_12.5_ P_25_ K_10_; ND—Nano DAP; NU—Nano Urea; NEB—Nano Emulsion Biofertilizer. Bars with different lowercase letters indicate significant differences among treatments (F-test, *p* ≤ 0.05). Error bars represent the standard error of the mean.

The findings demonstrated that NPK significantly improved chickpea GY (Figure 5I). The average GY of the NPK-fertilized chickpea was 13.54% greater than that of the NF item. When nano-emulsion biofertilizer was applied topically, a rise in GY was also noted. The 75% NPK variety showed a notable yield increase (21.63%) compared to the control, regardless of NPK fertilization. The production of chickpeas increased dramatically under foliar feeding, with variation and improved plant nutrition reaching a maximum of 1143.97 kg ha^−1^.

### 3.10 Stover yield

According to the study, chickpea plants’ SY was significantly impacted by the mineral fertilizers (Figure 5J). In contrast to locations where no mineral fertilizer was used, the SY increased by 73.28% in the 75% NPK applied variations. Compared with the ND and NU, the NEB significantly increased SY by 10.33 and 18.14%, respectively, regardless of NPK fertilization. The chickpea plants responded best to the NEB combination (Rhizobium + PSB) foliar spray in terms of SY. Compared to the other two foliar treatments, SY in this variety reached a maximum of 2024.09 kg ha^−1^, which was far higher.

### 3.11 Harvest index

The application of 75% NPK soil and NEB foliar applications has significantly influenced (*p* ≤ 0.05) HI (Figure 5K). The 75% NPK variant has shown a significant increase in HI of 48.07% compared to the control. Similarly, foliar application with NEB preparations resulted in significant increases of 21.50 and 44.81% compared to ND and NU, respectively.

### 3.12 Grain crude protein

**Figure 6:**
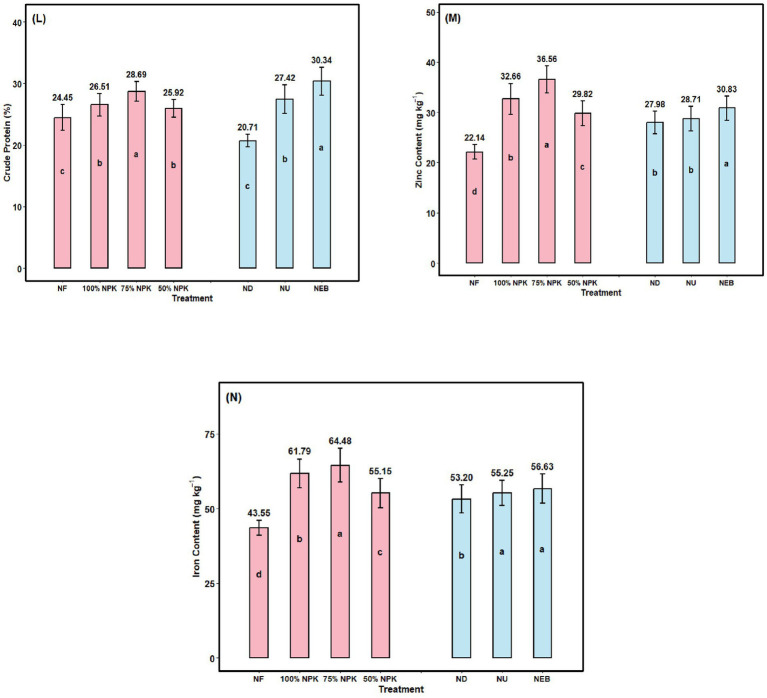
**(L)** Crude protein (CP), **(M)** Zinc content (Zn), and **(N)** Iron content (Fe) of chickpea depending on mineral fertilization, foliar application, and their combination; NF—N_0_ P_0_ K_0_; 100%NPK—N_25_ P_50_ K_20_; 75%NPK—N_18.75_ P_37.5_ K_15_; 50%NPK—N_12.5_ P_25_ K_10_; ND—Nano DAP; NU—Nano Urea; NEB—Nano Emulsion Biofertilizer. Bars with different lowercase letters indicate significant differences among treatments (*F*-test, *p* ≤ 0.05). Error bars represent the standard error of the mean.

Chickpea grain protein content was affected by the fertilizer treatments (Figure 6L). All fertilization treatments produced grain protein levels higher than the control. Maximum grain protein content (28.69%) was recorded in the 75% NPK variant, which was 17.34% higher than the control. Foliar sprays of NEB preparations contributed to a significant increase in grain protein content by 32.40% over the ND variant and by 46.50% over the NU variant.

### 3.13 Grain zinc content

The findings demonstrated that NPK significantly improved Zn content in chick pea grain (Figure 6M). Compared with the NF object, the 75% NPK increased by 65.13% across the NPK application variations. In a similar vein, the foliar application version showed a tendency to enhance Zn; however, the NEB variant showed a larger increase in Zn than the ND and NU, i.e., by 2.61 and 10.19%, respectively.

### 3.14 Grain iron content

The soil-applied 75% NPK and the NEB foliar application variant have significantly influenced grain iron content (Figure 6N). The 75% NPK variant has shown a significant increase in Fe of 48.06% compared to the NF. Similarly, foliar application with NEB preparations resulted in significant increases of 3.85 and 6.45% compared to ND and NU, respectively.

### 3.15 Data interpretation using Pearson correlation

**Figure 7:**
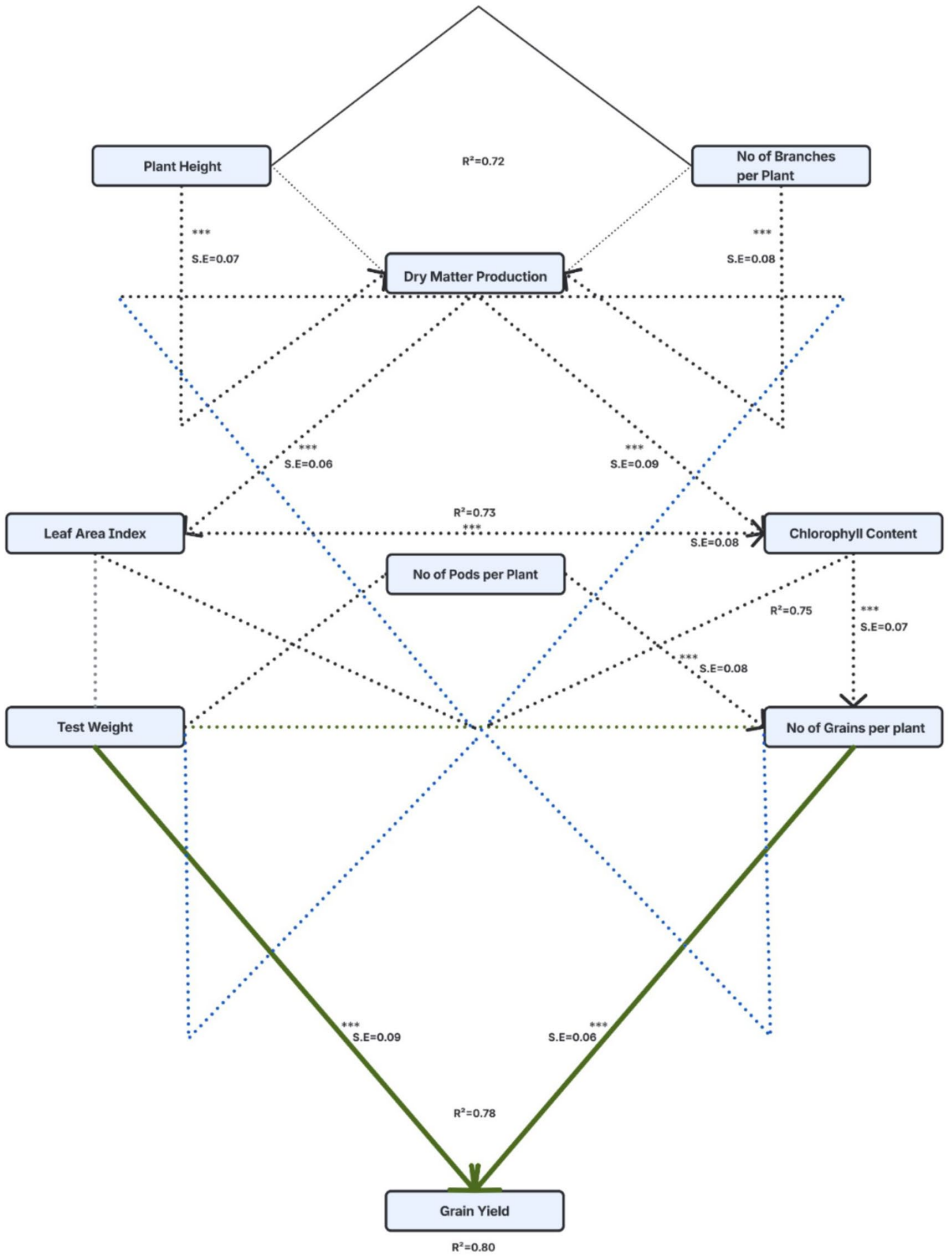
SEM analysis of the relationship between agronomic traits and yield in chickpea. The relationships among plant height (PH), number of branches per plant (NBP), dry matter production (DMP), leaf area index (LAI), chlorophyll content (CC), number of pods per plant (NP), number of grains per plant (NG), 100–seed weight (TSW), grain yield (GY), stover yield (SY), harvest index (HI), crude protein (CP), zinc content (Zn), iron content (Fe), * indicates significant level (*p* < 0.05); ** indicates very significant level (*p* < 0.01). *** indicates a very highly significant level (*p* < 0.001).

Research found statistically significant (*p* < 0.05) as well as highly statistically significant relationships (*p* < 0.01) between several of the variables measuring chickpea growth, dry matter, yield, yield attributes and quality attributes (Table 3). Results also demonstrated a positive correlation between the values of the mineral elements zinc and iron present in chickpea plants and the following growth characteristics: plant height (0.331*and 0.436**), number of branches per plant (0.326*and 0.348*), dry matter production (0.413**and 0.367*), leaf area index (0.228 and 0.231), chlorophyll (0.300*and 0.338*), number of pods per plant (0.425**and 0.355*), number of grains per plant (0.445**and 0.418**), 100-seeds weight (0.106 and 0.170), total grain yield (0.478**and 0.326*), total stover yield (0.927**and 0.889**) and harvest index (0.432**and 0.561*). Significant positive correlations were also found between the mineral contents of zinc and iron in protein (0.452 ** and 0.403*). The research also found that there is a highly significant (*p* < 0.01) positive correlation between total grain yield and the parameters that are related to growth, yield, and quality, including plant height (0.745**), number of branches per plant (0.780**), dry matter production (0.950**), leaf area index (0.474**), chlorophyll (0.869**), number of pods per plant (0.968**), number of grains per plant (0.892**), 100-seed weight (0.357*), and harvest index (0.530*). The correlation of yield in chickpeas with other quality parameters was also found to be significantly (*p* < 0.01) positive (0.478**and 0.326*; Figure 7).

**Table 3:** Pearson correlation coefficient of plant height.

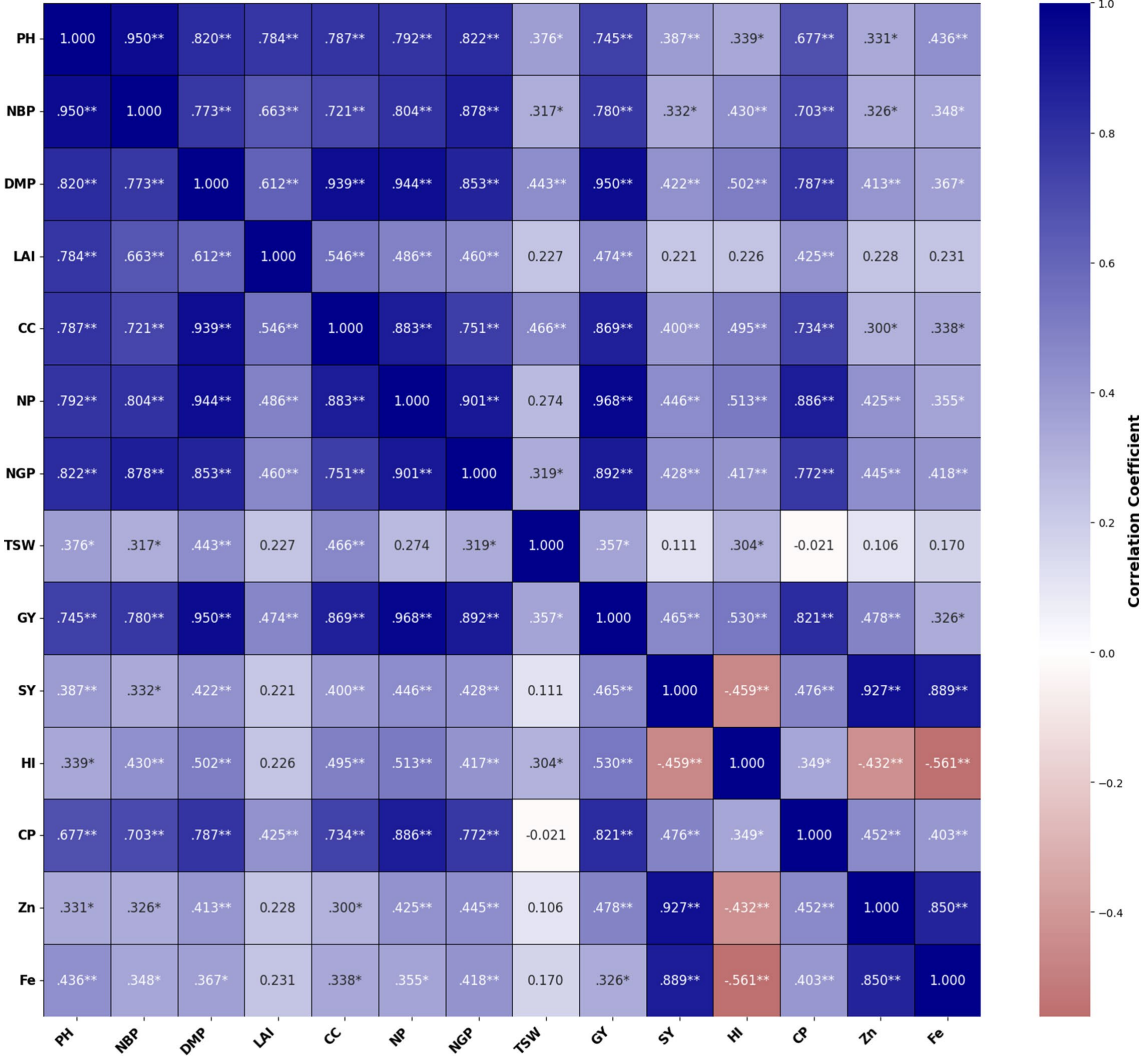

Plant height (PH), number of branches per plant (NBP), dry matter production (DMP), leaf area index (LAI), chlorophyll content (CC), number of pods per plant (NP), number of grains per plant (NG), 100—seed weight (TSW), grain yield (GY), stover yield (SY), harvest index (HI), crude protein (CP), zinc content (Zn), iron content (Fe); ns, * and ** represent no significant difference and significant differences at 5 and 1% probability levels, respectively.

### 3.16 Path analysis via structural equation modeling showing direct and indirect influences of major plant traits on yield in chickpea

Using a semi-structured questionnaire and agency records, this study employed structural equation modeling (SEM) to assess and clarify the direct and indirect effects of key traits of chickpea plants. By utilizing structural equation modeling (SEM), researchers were able to obtain a thorough understanding of how these treatments affected physiological and morphological characteristics of chickpeas in various paths both directly and indirectly to ultimately influence yield.

Using SEM analysis, it was also possible to identify the multitude of ways that treatment combinations influenced various agronomic characteristics (physiological and morphological) with respect to each other and ultimately to yield.

SEM provided an objective means of predicting the correlations between key characteristics impacting yield (R^2^ = 0.80) as well as a significantly positive correlation between the chickpea grain yield and important agronomic characteristics such as LAI, pods per plant, grains per plant and seed test weight. LAI appears to mediate the correlations between the aforementioned traits and grain yield, while also being directly affected by DMP. Thus, it is through the DMP traits (plant height and branch number) that primary growth characteristics create cascading effects on biomass production, larger plants, higher LAI and more pods, culminating in significantly higher yields. Although seed test weight does not have any impact on seed yields through other measurable traits in the proposed model, the direct impact of seed weight on yield is significant and highlights the critical nature of seed size/density in regards to chickpea seed yields. Chickpea yield is influenced by two factors; one is indirect [harvest index (HI) and chlorophyll content (CC)]. While both variables contribute to the ultimate chickpea yield through other factors, their impact is substantially less than that of the likely physiological processes related to canopy development and the resulting pattern of resource allocation, represented by the R-squared value (72% for DMP; 73% for LAI) for the variables tested. The latter emphasizes the importance of employing an optimal combination of traits (maximum vigor, minimum resource requirements and rapid growth rates) to increase potential yield in chickpeas. Findings from the study may facilitate the development of targeted breeding and agronomic strategies that result in increased yield through enhanced performance of these specific traits and optimal integration between them.

## 4 Discussion

### 4.1 Growth attributes of chickpea

The combined application of NPK and microbial fertilization showed superior crop growth performance, significantly improving plant height (PH), number of branches per plant (NBP), dry matter production (DMP), and leaf area index (LAI) in chickpea.

This study evaluated the impact of a nano-emulsion biofertilizer on both chickpea growth and development. The findings indicate that foliar applications of the same biofertilizer had beneficial effects regarding plant height, number of branches per plant, dry weight of all parts of each chickpea plant, leaf area index, and chlorophyll concentrations in all parts of the plant. The nano-scale size and higher surface area of these formulations facilitate rapid absorption and translocation of nutrients within plant tissues, thereby improving physiological processes such as photosynthesis and biomass accumulation. Similar improvements in plant height, branch number, and dry matter production in chickpea following nano-fertilizer application have been reported by Raliya et al. (2021) and Kumar et al. (2022). These findings further support the present results, where NEB foliar application promoted vegetative growth and physiological activity in chickpea plants. In addition to these benefits, foliar applications of the nano-emulsion biofertilizer increased the amount of nutrients available to plants through an alternative pathway compared to traditional soil-based fertilization methods, thereby improving nutrient absorbed by chickpea plants. Olanrewaju et al. (2017) found that when NPK fertilizer is combined with NPK-infused nano-emulsion developing bioproducts (biofertilizers), the treatment resulted in a synergistic effect that facilitated greater growth by chickpea plants as assessed by multiple parameters. The results of the studies show that when these two kinds of fertilizer are used together, it improves soil fertility significantly due to the increase in the amount of relative ease with which those nutrients, it enhances the physicochemical and biological functions of soil. This includes improving soil organic carbon content, aggregate stability, moisture-retention capacity, nutrient availability, and Activity of soil microbes (Selim, 2020). NPK, are critical nutrients for crop development, and deficiencies in any of these elements can adversely affect crop growth. Plant absorption through greater microbial dynamics in soil and root development, resulting in increased amounts of meristematic activity, which improved the ability of plants to take up the primary nutrients NPK at greater depths than with either of the fertilizers alone. Similar synergistic outcomes were observed by Bedada et al. (2014) and Bello et al. (2024), who found that NPK markedly enhanced crop yield. NPK fertilizers deliver a rapid source of macronutrients, ensuring immediate plant availability (Yadav et al., 2022).

This has resulted in the improvement in the height of the growth attributes of the plants that were into the soil treated with the dual combination of the two types of bio-chemical fertilizer (Ahmed et al., 2017; Singh et al., 2018; Kumar et al., 2018). Recent studies have also highlighted that nano-biofertilizers contain beneficial microorganisms that enhance rhizosphere activity and nutrient solubilization in legume crops. These formulations enhance nitrogen fixation, phosphorus solubilization, and root growth, thereby increasing nutrient acquisition and biomass accumulation. Growth traits have been reported under nano biofertilizer treatments in chickpea and other pulse crops (Singh et al., 2023; Meena B. L. et al., 2024). Such improvements are likely due to enhanced microbial colonization and improved nutrient cycling in the rhizosphere. Research results indicate a strong synergism between NPK and NEB with both of these parameters showing a marked improvement from that achieved by using NPK or NEB alone. Similar results were obtained using microorganisms and macro-fertilizer, as well as combinations of each type of fertilizer, for these same plant growth and yield parameters (Figure 3).

According to research, bio-fertilizer and chemical fertilizer have been shown to act positively together in regard to improving the efficiency of N and P in successively greater availability. This, in turn, contributed to increased growth. The researchers said that the increased growth attributes were due to improved photosynthesis and increased movement of photosynthetic products from sources to storage sites (Sodavadiya et al., 2023; Parmar et al., 2023). According to Srivastava et al. (2024), the improvement is primarily associated with increased availability of fertilizer in the soil and improving nanotechnology to provide nutrients through foliar sprays of bio-fertilizers. The improved availability of the requisite nutrients has positively affected cell division and elongation, thereby favorably impacting the growth component of green gram crops throughout their life cycle. Albayrak et al. (2006) reported that Microorganisms have a positive influence on plant growth by producing a plant hormone (auxin) that is manufactured by the Rhizobium species of bacteria as a secondary product in plants inoculated with rhizobium. Plant growth-promoting rhizobacteria are also known to produce phytohormones such as indole-3-acetic acid (IAA), gibberellins and cytokinin, which stimulate root elongation and lateral root formation. These hormones enhance root surface area and improve the plant’s ability to absorb water and nutrients from the soil. Consequently, enhanced root architecture leads to greater biomass accumulation and improved vegetative growth in legume crops. The enhanced plant height, number of branches, and dry matter production observed in the present study may therefore be attributed to improved root growth and hormone-mediated stimulation of plant development associated with microbial biofertilizer application (Fasusi et al., 2021). As a result of the increased carbohydrate conversion rate into protein, the downstream products of carbohydrate conversion (i.e., protoplasm and cell wall materials) were synthesized at increased levels. Furthermore, the increase in cell size was reflected morphologically in taller plants; consequently, there was greater accumulation of dry matter due to the larger cells (Babubhai, 2020). Inoculation with nano-biofertilizers has been shown to increase root nodulation, which may lead to greater root development, improved nutrient availability, and rapid plant vigor, resulting in greater dry matter accumulation and improved flowering and pod development (Meena et al., 2013; Patil et al., 2012; Devakumar et al., 2014). Increased dry matter production is due to balanced amount of macro and micro nutrients through foliar fertilization which resulted in better crop growth and photosynthetic activity which led to better supply of photosynthates ultimately resulted in higher dry matter production per plant (Dass et al., 2022). Subba Rao (1993) stated that the favorable effect of biofertilizers on growth parameters might be ascribed to its important role in fixing atmospheric N as well as increasing the secretion of natural hormones, namely, IAA, GA3, and cytokinin, antibiotic, and possibly raising the availability of various nutrients.

The application of nitrogen fertilizer has been shown to delay leaf senescence as well as enhance the number of leaves produced per plant and the Leaf Area Index (LAI) produced by the plant. Escalante-Estrada et al. (2014) also established that mineral nutrient availability, particularly nitrogen, is a large contributor to the number of leaves produced by the plant and how quickly New Leaf Blades (NLBs) emerge from the plant. With adequate nitrogen, leaf senescence is delayed, and the duration of time that each leaf produces photo assimilates (photo assimilate accumulation period) increases, thereby producing significantly more photo assimilates and transferring them to the grain. Consistent with these results, Namvar et al. (2011) also observed that a greater amount of Leaf Area was produced when higher amounts of nitrogen fertilizer were applied to the plant and that this was largely due to an increase in the number of leaves produced per plant.

In chickpeas, the use of an NPK, was found to improve the leaf surface area measurements in comparison to the control treatment. Microbial inoculants and micro fertilizers had no significant effect on leaf expansion; however, both NPK with microbial application leaf development and the synthesis of organic compounds, with NPK having the greatest effect. According to Thalooth et al. (2006), the stimulatory effects of trace metals on several biological and metabolic processes in plants accelerate enzymatic activity, increase leaf blade expansion, and promote the accumulation of photosynthetic pigments.

In an investigation by Yang et al. (2009), inoculating chickpea plants with beneficial microbes enhances nutrient absorbed, even during periods of salt stress, and subsequently promotes the formation of chlorophyll pigments. The increased capacity of the chickpea plant to uptake residual nitrogen from its combination with chemical fertilizers promotes longer-term nutrient supply to the plant. Our results for the NEB treatments match those of the studies by Priya et al. (2021) and Abhirami et al. (2023), which also showed that high nitrogen concentrations increase chlorophyll concentrations.

The increased chlorophyll content observed under nano-emulsion biofertilizer treatments could be associated with improved nutrient uptake and enhanced physiological activity in plants. Biofertilizers containing beneficial microorganisms improve nitrogen availability, a key component of chlorophyll molecules and an essential factor in photosynthesis. Enhanced nutrient availability promotes chlorophyll synthesis, increases photosynthetic efficiency, and ultimately improves growth attributes. Similar improvements in chlorophyll content and photosynthetic activity following PGPR application have been reported under various environmental conditions (Batool et al., 2020). Chlorophyll is one of the primary pigments used by plants to absorb light energy for photosynthesis and is responsible for the green color of greens on green gram plants. Similarly, recent research indicates that nano-nutrient formulations improve chlorophyll synthesis and photosynthetic efficiency by enhancing the availability and assimilation of essential nutrients such as nitrogen, magnesium, and iron. Increased chlorophyll content and improved photosynthetic performance have been reported in legume crops following foliar application of nano-fertilizers due to their ability to facilitate efficient nutrient transport and metabolic activity (Dimkpa and Bindraban, 2021; Raliya et al., 2021). These mechanisms may explain the higher chlorophyll concentration observed in chickpea plants under NEB treatments in the present study.

### 4.2 Yield attributes of chickpea

NPK mineral fertilizers with microbial foliar fertilization have significantly improved yields of chickpeas and their structural components, viz., number of pods (NP), the number of grains (NG), the 100-grain weight (TSW), and seed yields (SY), with N_18.75_ P_37.5_ K_15_ being twice as effective as an unfertilized control plant (N_0_ P_0_ K_0_). This study supports previous findings from Sahu et al. (2020). They also noted that NPK fertilizers improved plant growth, pod and seed numbers per plant, and the overall SY of chickpeas. NPK integration improves the efficiency of nutrient usage, which increases chickpea yield. Applying nitrogen to plants in their early growth stages promotes vegetative growth, which creates ideal circumstances for large harvests. Plant protein levels and overall yield are directly increased by nitrogen, which is essential for the production of proteins and chlorophyll. Phosphorus is essential for plant metabolism and energy generation, flowering, pod development and blooming. Applying potassium to chickpeas improved the harvest’s quantity and quality. By providing essential nutrients that enhanced yield metrics and increased seed yield, the integration of chemical fertilizers created a favorable soil condition (Isha et al., 2024).

The combination of these two types of fertilizer has provided a variety of different results in relation to more successful measurements such as increased number of pods per plant, increased number of grains per pod, increased weight of grain (100 grams), increased grain yield, increased stover yield, and a higher harvest index. The genetic variation among different varieties of chickpeas explains the variation in pod number and grain number per pod, primarily due to increased branch formation, which has been shown to have a positive correlation with pod number (Sarvajeet et al., 2018). The observed improvement in yield and nutrients absorbed by plants can be primarily attributed to the optimized application of NPK fertilizers, which enhanced nutrient availability within the rhizosphere of chickpea. Balanced fertilization promoted robust root system development, facilitating efficient nutrient absorption by plants and significantly increasing NPK absorption by plants. Mainly P contributed to enzymatic activation, osmotic balance, and efficient translocation of photo assimilates (Murthy et al., 2024).

Moreover, the synchronized supply of essential nutrients ensured their availability during critical growth stages, thereby improving nutrient use efficiency and reducing potential losses. Enhanced uptake of N and P stimulated photosynthetic activity and assimilate partitioning toward reproductive structures, resulting in increased grain yield and biomass accumulation. The positive correlation between fertilization application rates and crop productivity the effectiveness of balanced NPK fertilization in achieving higher yield targets (Bhavya and Basavaraja, 2021). Nie et al., 2018, observed that long-term NPK application in paddy soils reduced fungal diversity without altering bacterial diversity. This pattern likely reflects ecological differentiation chemical fertilizers deliver readily available macronutrients that favor r-strategist, fast-growing bacterial taxa Averill and Waring (2018), while fungi (key decomposers of complex organic matter) may be disadvantaged by the reduced carbon inputs and accelerated nutrient cycling typical of high-NPK systems (Frey et al., 2014).

Fertilizer use in this study produced an 11.56% increase in seed yield from the full-dose application (N_25_P_50_K_20_) compared to the unfertilized control (N_0_P_0_K_0_) and a 7.42% increase from the half-dose application (N_12.5_P_25_K_10_). Past research has shown that adding mineral nitrogen encourages the development and growth of plants, in addition to as well as increasing crop production, even in arid climates; in the current research, however, the climate conditions throughout the 2023 chickpea growing season were not optimal due mainly to lack of rain in August and September (Figure 2). While 2023 and 2024 yield reductions have been significant, the effect of climate on chickpeas grown in 2023 was less detrimental due to favorable soil conditions, especially the low-humus, silty clay loam with 5.15% humus, which allowed moisture to be stored effectively over a longer period. Soil organic matter plays a crucial role in improving soil structure by promoting aggregate formation, thereby enhancing water infiltration, moisture retention, and aeration conditions essential for optimal root growth. It also functions as a reservoir of key nutrients, including N and P, which are gradually released through microbial decomposition and mineralization processes. Furthermore, higher Soil organic matter levels have been shown to positively correlate with improved nutrient use efficiency and increased crop productivity across both conventional and conservation agriculture systems (Young et al., 2021). Salvagiotti et al. (2008) reviewed studies from 1966 to 2006 and found that mineral nitrogen fertilization boosted soybean height, dry matter accumulation, lateral branch development, pod and seed numbers, resulting in higher biological yields. Additionally, the analysis showed that for each additional one (1) kilogram of nitrogen in the aboveground biomass, the seed yield increased by an average of 0.013 mg.

The proportions of each nutrient in a fertilization strategy can affect chickpea yields. The highest average seed yield was recorded by Saeed et al. (2004) at 2.37 tons per hectare (t ha-1), for the N_35_P_87.5_K_100_ treatment. The yield also decreased when the amounts of each of the three nutrients were proportionately increased or decreased. Shan et al. (2016) reported that applying N_30_P_60_K_30_ mineral fertilizers significantly increased crop yield to 5.46 t ha^−1^ and 100-seed weight to 237 g, outperforming the N_30_P_60_K_00_ treatment (4.26 t ha^−1^ and 230 g). At the 75% NPK level, the hybrid Heinz 9,553 exhibited the highest productivity (191.39 t ha^−1^), significantly outperforming the other hybrids. In contrast, at the full fertilizer dose (100% NPK), the overall mean productivity declined to 150.26 t ha^−1^. Under this condition, the hybrids BHN (182.15 t ha^−1^) and Heinz (155.38 t ha^−1^) performed better than BRS Sena, which recorded the lowest yield (113.23 t ha^−1^) in tomato plant was recorded by de Almeida Neta et al. (2020).

The combination of foliar applications of plant growth regulators (PGRs) with rhizobium and phosphate-solubilizing bacteria significantly increased chickpea yields compared to the control treatments. This combination enhanced not only flowering and pod set but also increased harvest index and biomass accumulation, thereby increasing chickpea yield (Rafique et al., 2021). Therefore, combining foliar applications of PGRs with beneficial microbes is an efficient way to improve the performance of leguminous crop species. The increase in yield characteristics and yield of chickpea can be attributed to an increase in available nutrients, thereby increasing the synthesis of chlorophyll in leaves because bio-fertilizers have significant magnesium content along with other nutrients, which aided in generating higher values for yield characteristics (Verma et al., 2020; Rolaniya et al., 2023). Dutta and Bandyopadhyay (2009) demonstrated that reducing phosphorus fertilization by up to one-third in chickpea, when combined with inoculation of P-solubilizing *Pseudomonas* sp., did not adversely affect plant growth and development parameters. This effect can be attributed to the enhanced availability of phosphorus through microbial solubilization processes, which convert insoluble forms of phosphorus into plant-accessible forms, thereby compensating for the reduced fertilizer input. The combined application of microbial inoculants with reduced rates of mineral NPK fertilizers significantly enhanced the yield of maize and wheat compared to the application of reduced NPK fertilizers alone in the present study. This improvement can be attributed to enhanced nutrient use efficiency mediated by microbial activity, including increased nutrient solubilization, mineralization, and improved root uptake. Microbial inoculants are known to stimulate plant growth through multiple mechanisms such as phytohormone production and improved rhizosphere interactions, thereby compensating for reduced fertilizer inputs (Bhardwaj et al., 2014). In agreement with these findings, Oliveira et al., 2017 reported that inoculated maize plants grown under an 80% reduction in nitrogen fertilizer achieved yields comparable to those under full fertilization, highlighting the potential of microbial-assisted nutrient management strategies.

In this study, macro fertilizer application (S) significantly improved the number of pods (NP), grains per plant (NG), thousand-seed weight (TSW), seed yield (SY), and harvest index (HI) relative to the control, as shown in Figures 4, 5. Foliar fertilization of plants with nutrients is an effective way to fertilize because it reduces nutrient loss through the soil, enhances the volume and quality of the food produced, and makes nutrients more readily available for plant absorption. The additive benefit of combining foliar applications of mineral fertilizers with soluble fertilizer biofertilizer has previously been established by Gul et al. (2011). The findings in this study are consistent with those stated previously: The use of NEB (plant-bacteria) for microbial foliar application in conjunction with a macro fertilizer is a good way of helping to increase yield and general productivity of chick peas. Nano-fertilizers enhance nutrient availability and uptake due to their high surface area and better mobility within plant tissues, resulting in improved physiological activity and biomass accumulation. Similar increases in pod number, seed weight and grain yield were reported in chickpea when nano-nutrient formulations were integrated with conventional fertilization strategies (Raliya et al., 2021; Kumar et al., 2022). The microbial inoculation provides many advantages to soil through the increase of the biological activity of the soil, as well as improving the efficiency of nutrient absorbed.

Research has demonstrated that the use of beneficial nitrogen-fixing and phosphorus-solubilizing microorganisms, together, can significantly increase chickpea seed nitrogen among other characteristics that cause an increase in seed quality. Mohammadi et al. (2010) found that combining these beneficial microorganisms resulted in a significantly improved quantity and quality of chickpeas produced. Rudresh et al. (2005) also found a significant increase in both the quality and quantity of chickpeas produced with the combined application of beneficial microorganisms. Kheroar et al. (2018) showed that the use of biofertilizers improves plant resistance to abiotic stress. Biofertilizers were found to stimulate root and shoot biomass, increase the number of tillers, increase grain weight, and allow for greater nutrient absorbed by the crop. This effect on the rice crop resulted in greater yields, while Meena et al. (2020) also found that combining mineral fertilizers with microbial inoculant treatments provided the most beneficial means of maximizing chickpea productivity.

The superior performance observed with 75% of NPK and nano emulsion biofertilizer (NEB) relates to increased use efficiency of nutrient supply through increased activity in the rhizosphere. Also, lower levels of fertilizer will decrease nutrient losses through leaching and volatilization and increase nutrient retention and plant uptake efficiency (Cassman et al., 2002; Zhang et al., 2015). Increased microbial mediated processes (e.g., nutrient solubilization, mineralization, and biological fixing of nitrogen) as a result of the use of biofertilizers can also increase the availability of nutrients (Vessey, 2003; Richardson et al., 2009). Using a nano-emulsion formulation increases the efficacy of microbial inoculants by ensuring they are stable and dispersed within a soil environment, which can increase root colonization and prolong activity in the rhizosphere (Kah et al., 2018). Therefore, the synergy created between lower chemical inputs and biological amendments is likely to have contributed to an increased output of crops compared to using full levels of NPK. Conversely, an over-application of fertilizer (100% NPK) could cause imbalances of nutrients and have negative interactions between these inputs, resulting in lower efficiency of nutrient utilized (Fageria, 2001; Rietra et al., 2017). Previous studies have shown that replacing part of chemical fertilizer with microbial inoculum can help maintain or increase crop yields. For example, (Oliveira et al., 2018) found that maize grown with reduced N levels produced similar yields as those grown with full rates of N when inoculated with beneficial bacteria.

### 4.3 Grain quality contents

The researchers showed that all treatments with 75% NPK and differing NEB foliar treatments greatly improved the quality of protein and also improved grain quality in chickpeas. The results presented here are consistent with findings reported by Patil et al. (2012) and Pingoliya et al. (2015) with pea (*Pisum sativum* L) and chickpeas (Khafagy et al., 2019). Upon further examination, nutrient availability for N, P, and K was optimal to improve plant growth through an increase in the rate of photosynthesis, which leads to a greater protein content of the plant (El-Azab, 2016). It is hypothesized that NEB promotes plant development by increasing nutrient availability and uptake of chickpea by colonization of the rhizosphere, which ultimately results in higher levels of protein content in chickpea (Yildirim et al., 2011; Ejaz et al., 2020; Ali et al., 2020). Nano based nutrient formulations improve nutrient solubility, transport and assimilation within plant tissues, thereby increasing protein synthesis and micronutrient accumulation in seeds. Enhanced zinc and iron concentrations in legume grains under nano fertilizer treatments have been reported due to improved nutrient uptake efficiency and translocation from vegetative tissues to developing seeds (Singh et al., 2023; Meena D. et al., 2024; Meena B. L. et al., 2024).

Higher grain zinc levels (75% NPK: 36.56 mg kg^−1^) caused by increased nutrient uptake through an increased availability of zinc in the root zone, increased efficiency of absorption and increased cation-exchange capacity of roots, because of the foliar application of NEB (30.83 mg kg^−1^). Zinc also promotes key physiological and metabolic activities in plants. In contrast, the recommended NPK dose alone resulted in significantly reduced seed zinc content (22.14 mg kg^−1^), attributable in part to limited zinc in the root zone and diminished overall nutrient absorption by the crop. According to research, the application of N increases the amount of Zn absorbed by the roots, moved from the roots to the shoots, and redistributed from the source to the sink tissues (Erenoglu et al., 2011). Similarly, N fertilization increased Zn levels in both seeds, shoots, and roots; therefore, this agrees with the findings of the present study (Olama et al., 2014). According to Pal et al. (2019), when applied to chickpea crops, urea fertilizer increased the amount of zinc found in both the plant’s grain and shoot significantly. Similarly, Zhao et al. (2022) analysis of various studies done globally showed significant elevations in zinc concentrations found within wheat, maize, and rice grains when grown with N Fertilizer as opposed to those grown without fertilization. As a result of improved (Fe) availability either through the application of soil or Foliar induced Fe, as well as an increased concentration of Fe stored in the soil, and through successful penetration of Foliar applied Fe into the leaf, either through translocation or stomatal absorption, the Fe reserves in seeds from the above treatments are much higher than those from other treatments. Conversely, chickpea seeds in the control plots (43.55 mg kg^−1^) showed markedly lower Fe levels, likely due to inadequate soil nutrient status, which restricted overall nutrient uptake. Comparable findings have been reported by, Gowthami and Ananda (2017) in groundnut, and Hanumanthappa et al. (2018) in pigeon pea.

## 5 Conclusion

The combined application of reduced basal NPK fertilizers with foliar sprays of nano-emulsion biofertilizers demonstrated clear superiority in improving chickpea performance across growth, yield, and grain quality parameters. This integrated approach markedly enhanced vegetative characteristics, including plant height, number of branches, biomass accumulation, leaf area index, and chlorophyll concentration. Simultaneously, it improved reproductive attributes such as pod formation, seed number per plant, test weight, overall grain and stover yield, harvest index, and nutritional quality traits like protein, zinc, and iron content.

From a practical standpoint, the most effective and economically viable treatment for semi-arid regions with silty clay loam soils involves applying 75% of the recommended NPK dose (e.g., N_18.75_, P_37.5,_ K_15_ kg ha^−1^ as basal) combined with foliar application of nano-emulsion biofertilizers at 10 mL L^−1^ during 30 and 45 days after sowing. This strategy reduces chemical fertilizer usage by 25% while maintaining or even enhancing productivity and profitability. Additionally, seed treatment with nano-emulsion biofertilizers, though low in cost, significantly boosts Microbial dynamics in soil and nutrient availability, making it a practical and scalable option for smallholder farmers. Overall, this integrated nutrient management approach supports sustainable chickpea cultivation by reducing dependency on synthetic fertilizers, minimizing environmental losses, enhancing soil biological health, and improving grain nutritional quality. Its long-term adoption holds promise for increasing resilience in pulse-based systems while addressing both economic and nutritional security under changing climatic conditions.

## Data Availability

The original contributions presented in the study are included in the article/supplementary material, further inquiries can be directed to the corresponding author.
